# Inflammatory cytokine levels in synovial fluid 3, 4 days postoperatively and its correlation with early-phase functional recovery after anterior cruciate ligament reconstruction: a cohort study

**DOI:** 10.1186/s40634-016-0067-z

**Published:** 2016-11-02

**Authors:** Makiko Inoue, Takeshi Muneta, Miyoko Ojima, Kaori Nakamura, Hideyuki Koga, Ichiro Sekiya, Mutsumi Okazaki, Kunikazu Tsuji

**Affiliations:** 1 Department of Plastic and Reconstructive Surgery, Graduate School of Medical Sciences, Tokyo Medical and Dental University, 1-5-45 Yushima, Bunkyo-ku, Tokyo, 113-8510 Japan; 2 Department of Joint Surgery and Sports Medicine, Graduate School of Medical Sciences, Tokyo Medical and Dental University, 1-5-45 Yushima, Bunkyo-ku, Tokyo, 113-8510 Japan; 3 Center for Stem Cell and Regenerative Medicine, Tokyo Medical and Dental University, 1-5-45 Yushima, Bunkyo-ku, Tokyo, 113-8510 Japan; 4 Department of Cartilage Regeneration, Graduate School of Medical Sciences, Tokyo Medical and Dental University, 1-5-45 Yushima, Bunkyo-ku, Tokyo, 113-8510 Japan

**Keywords:** Anterior cruciate ligament, Synovial fluid, Inflammatory cytokines, Recovery after surgery, Preoperative period

## Abstract

**Background:**

Synovial fluid was collected prior to and at 3 to 4 days after ACL reconstruction to investigate the correlation between inflammatory cytokine levels in the acute phase after surgery and physical functional recovery at 3 months postoperatively.

**Methods:**

For this purpose, 79 patients with ACL reconstruction using semitendinosus tendons were included in the study. Median days from injury to surgery were 80 days (13–291 days). Synovial fluid was obtained just before surgery and at 3 to 4 days after surgery. Physical activity of each patient was evaluated at 3 months postoperatively, and scored from 0 (hard to walk) to 5 (run). Patients able to jog (score 4) or run (score 5) were considered as the “quick recovery” group and others (scores 1–3) as the “delayed recovery” group.

**Results:**

Physical activity recovery scores in the early surgery group (preoperative period less than 60 days; Group I) were significantly better than those in the delayed surgery group (Group II). Among the cytokines tested, TNF-alpha and IL10 levels in synovial fluid were significantly higher in Group II at 3 to 4 days postoperatively, while levels of these cytokines were quite comparable preoperatively between the groups. Increased IL1-beta expression was noted in the delayed recovery group at 3 to 4 days postoperatively. In addition, levels of IL6, IL10 and IFN-gamma also tended to increase in patients with delayed recovery.

**Conclusion:**

Delayed ACL reconstruction increases levels of inflammatory cytokines in synovial fluid after surgery and correlates with a prolonged recovery of short-period physical activity of the patients.

## Background

The anterior cruciate ligament (ACL) graft undergoes a long-term healing process known as “ligamentization” (Claes et al. 2011; Janssen & Scheffler 2014; Scheffler et al. 2008). It will be important for surgeons to classify the variation of the healing process after surgery in individual patients.

A review article regarding the return to sports after ACL reconstruction revealed that on average, 81 % of patients returned to any sport, 65 % returned to their pre-injury level of sport, and 55 % returned to a competitive level sport after surgery (Ardern et al. 2014; Petersen et al. 2014). Age, preoperative period, inflammatory reaction and pain are considered to affect the functional recovery after ACL reconstruction from the early phase (Czuppon et al. 2014; Feller & Webster 2013; Petersen et al. 2014). A systematic review classified the injury-to-surgery interval as either early (ranging from within 2 days to 7 months) or delayed (ranging from 3 weeks to 24 years) and found that 8 articles recommended early reconstruction, whereas the majority of the literature found no difference in outcome between early and delayed surgery (Andernord et al. 2013). These indicate that the effect of the timing of surgery after injury is still controversy (Krutsch et al. 2015; Kwok et al. 2013).

“Synovial fluid has been extensively investigated for joint degeneration-related markers upon ACL injury and after ACL reconstruction surgery” (Åhlén et al. 2015; Harkey et al. 2015; Mendias et al. 2013). Bigoni, et al. reported increased levels of pro-inflammatory cytokines (IL-6 and IL-8) in the acute phase of inflammation following ACL injury (Bigoni et al. 2013). Our previous study suggested that osteopontin levels in synovial fluid are associated with the severity of joint pain and cartilage degradation after ACL rupture (Yamaga et al. 2012).

The purpose of this study was to measure inflammatory cytokines in synovial fluid preoperatively and at 3 to 4 days after ACL reconstruction surgery and to investigate the correlation between each cytokine and the functional recovery status at 3 months postoperatively. The hypothesis of this study was that the levels of inflammatory cytokines in synovial fluid are inversely correlated with the functional recovery of the patient at 3 months after surgery.

## Methods

### Patients

This study was approved by the Ethics Committee of Tokyo Medical and Dental University. The approval number was 455. All patients included in this study gave their full written, informed consent for participation in this study prior to the ACL reconstruction surgery. Synovial fluid was obtained just before surgery and at 3 to 4 days after surgery.

Seventy-nine patients who underwent ACL reconstruction from January 2013 through April 2014 were included in this study (female: 40, male: 39; 12–62 year-old, median 19 year-old; average Tegner score 7.0+/−0.9, Table 1). Median days from injury until index surgery were 80 days (13–291 days). Ninety-six percent (76 out of 79) of patients did not have any knee injury on the contralateral side. Patients with a preoperative period of less than 60 days were defined as the early surgery group (Group I), while patients with a preoperative period 60 days and longer were defined as the delayed surgery group (Group II). All surgeries and follow-ups were conducted by surgeons of the knee and sports medicine group in our university hospital.

**Table 1:** Patient information and clinical outcomes

	Total	Group I (Preoperative periods < 60 days)	Group II (Preoperative periods ≥ 60 days)	*P* value
Number of patients	79	27	52	
Periods from injury until index surgery (day)	80 (13–291)	44 (13–58)	119 (60–291)	
Median (range)				
Age (year)	19 (12–62)	21 (14–52)	19 (12–62)	0.84
Median (range)				
Gender (M;F)	39;40	15;12	25;27	0.63
Tegner score	7.0 ± 0.9	7.3 ± 0.8	6.9 ± 0.9	0.10
Average ± SD				
Meniscus injury	28 (35.4 %)	10 (37.0 %)	18 (34.6 %)	1
Recovery score	3.2 ± 0.8	3.4 ± 0.7	3.1 ± 0.8	0.04
Average ± SD				

### Surgical technique

An anatomic double-bundle technique using two autologous double-stranded semitendinosus tendons was employed for a remnant-preserving ACL reconstruction by the behind-remnant approach (Muneta et al. 2015). Tibial tunnels were created in the original remnant and just lateral to the medial tibial eminence. Fixation was achieved by pull-out technique using two EndoButtons CL BTB (Smith & Nephew, Andover, FL), and two anchor staples (Meira corporation, Aichi, Japan). Three #2 strong sutures were used for tibial fixation. The initial tension was set proportionally to the graft diameter at 20° flexion (Koga et al. 2015).

### Postoperative management

The patients followed a rehabilitation protocol described before (Muneta et al. 2015). The patients were encouraged to initiate knee range motion from the 3^rd^ day postoperatively. Patients were allowed to bear weight using an immobilized brace (Knee Brace®, Alcare, Tokyo, Japan) from the 2^nd^ day postoperatively as tolerated. Usually, braces and crutches were no longer used after 4 weeks postoperatively. Jogging was allowed at 2.5 months post-surgery when a single-leg half squat was achieved safely on the injured leg. The patients were encouraged to practice specialized training for their intended athletic activities after 80 % of their full running capacity was achieved. The patients were expected to return to their pre-injury athletic activities 6 months or more after surgery based on individual recovery of athletic performance.

### Scoring of physical activity at 3 months after ACL reconstruction

Physical activity of each patient at 3 months after surgery was evaluated and scored according to the criteria indicated in Table 2. Patients who can jog (score 4) or run (score 5) were classified as the “quick recovery” group, and others (scores 1, 2, or 3) were classified as the “delayed recovery” group. Jogging was allowed after 2.5 months postoperatively if the patient could perform single-leg squatting easily on each limb.

**Table 2:** Physical activity at 3 months post surgery

Score	Activity	Category
1	Hard to walk	Delayed recovery
2	Walking	
3	Stair climbing	
4	Jogging	Quick recovery
5	Running	

### Cytokine quantitation

Cytokine levels in synovial fluid were quantitated by multiplex ELISA system (V-PLEX human pro-inflammatory panel, Meso Scale Discovery, MD). Levels of TNF-alpha, IL1-beta, IL2, IL6, IL8, IL10, and IFN-gamma in synovial fluid were quantitated according to the manufacturer’s protocol.

### Statistical analysis

EZR was employed for statistical analyses (Kanda 2013). Student’s *t*-test, Mann-Whitney’s *U*-test, Fisher’s exact test, and the Jonckheere- Terpstra trend test were employed for the statistical analyses in this study. Prior to statistical analyses, the Smirnov-Grubbs test was performed to exclude outliers. *P* values less than 0.05 were considered as significant.

## Results

### Early and delayed surgery groups

**Fig. 1:**
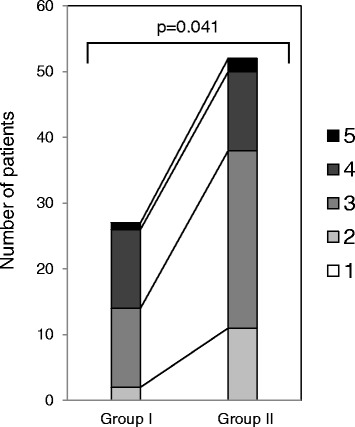
Frequency distribution of the 5-grade of physical activity score in quick and delayed recovery groups. The physical recovery score of Group I and Group II were evaluated according to the criteria indicated in Table 1. Mann-Whitney’s *U*-test was employed for statistical analysis. Group I; patients with preoperative periods less than 60 days. Group II; patients with preoperative periods of 60 days or longer

According to the 5-grade of physical activity score, the score of Group I was significantly better than that of Group II (Fig. 1). No significant differences in age (Student’s *t*-test), gender (Fisher’s exact test), Tegner score (Mann-Whitney’s *U* test), and injury history (Fisher’s exact test) were observed between the groups (Table 1).

### Cytokine levels in synovial fluid preoperative and at 3 to 4 days after surgery

Prior to ACL reconstruction, it was impossible to obtain synovial fluid by arthrocentasis in half of the patients because of the small volume accumulation in the joint. In all patients, synovial fluid volume surged 3 to 4 postoperatively (average 27+/−12.5 ml).

**Fig. 2:**
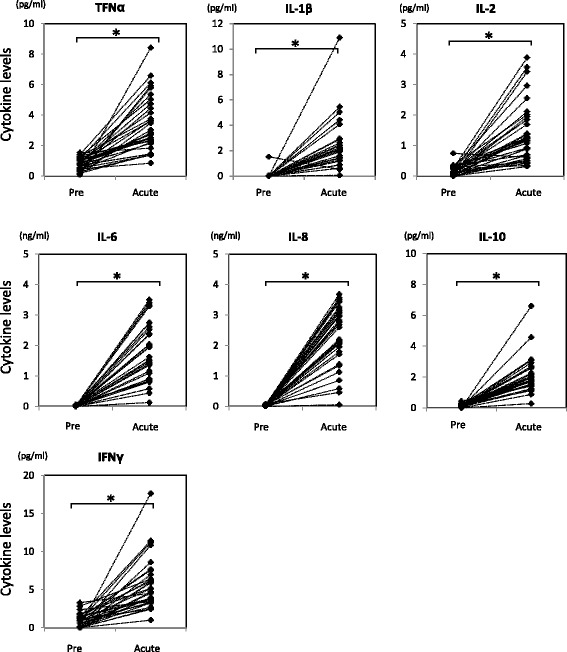
Levels of cytokines in synovial fluid were significantly increased after index ACL reconstructive surgery. Cytokine levels in synovial fluid were quantitated by Multiplex ELISA. Student’s *t*-test was employed for statistical analysis (Asterisk indicates *p*-value less than 0.05). Pre; synovial fluid harvested at surgery. Acute; synovial fluid harvested at 3 to 4 days after surgery

Multiplex ELISA indicated that the levels of TNF alpha, IL1 beta, IL2, IL6, IL8, IL10, and IFN gamma were significantly increased at 3 to 4 days after surgery in comparison to preoperative levels, although the degree of the increased expression level of each cytokine varied in each patient (Fig. 2).

### Levels of TNF alpha and IL10 in synovial fluid at 3 to 4 days after surgery were significantly increased in delayed surgery group

**Fig. 3:**
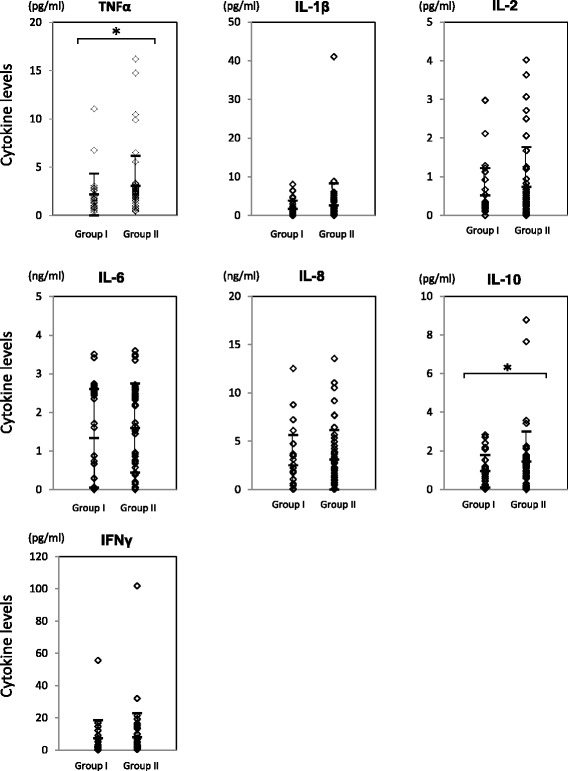
Levels of cytokines in synovial fluid at 3 to 4 days post ACL reconstruction surgery. Cytokine levels in synovial fluid at 3 to 4 days post ACL reconstruction surgery were quantitated by Multiplex ELISA. The Mann-Whitney’s *U*-test was employed for statistical analysis (Asterisk indicates *p*-value less than 0.05). Group I; patients with preoperative periods less than 60 days. Group II; patients with preoperative periods of 60 days or longer

**Fig. 4:**
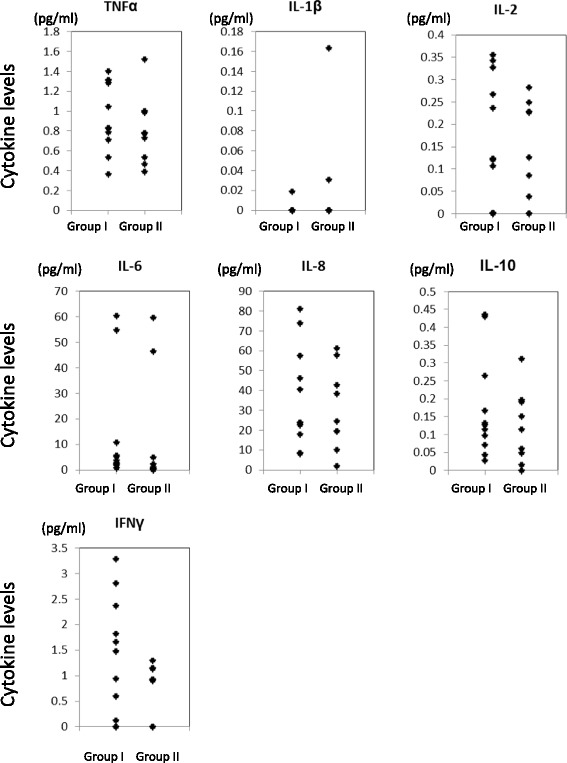
Levels of cytokines in synovial fluid before ACL reconstruction surgery. Cytokine levels in synovial fluid before ACL reconstruction surgery were quantitated by Multiplex ELISA. The Mann-Whitney’s *U*-test was employed for statistical analysis (Asterisk indicates *p*-value less than 0.05). Group I; patients with preoperative periods less than 60 days (*n* = 13). Group II; patients with preoperative periods of 60 days or longer (*n* = 11

Among the cytokines tested, TNF alpha and IL10 levels in synovial fluid were significantly higher in Group II (Fig. 3), while the levels of these cytokines prior to index surgery were quite comparable among the patients in both Group I and Group II (Fig. 4).

### Physical activity recovery at 3 months and inflammatory cytokines at 3 to 4 days postoperatively

**Fig. 5:**
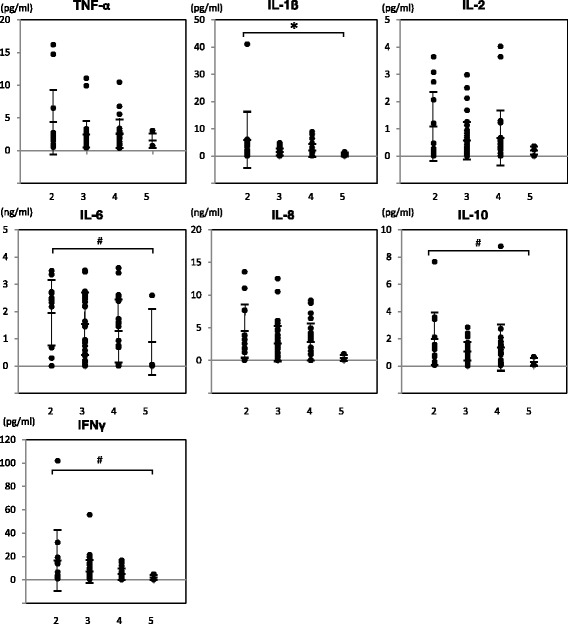
Increased levels of IL1 beta correlated with hindrance of patients’ physical activity at 3 months. Jonckheere-Terpstra test was performed to evaluate if the levels of each cytokine were correlated with the patients’ degree of physical activity at 3 months (*: *p* = 0.03, #: *p* = 0.08). X-axis; patients’ 5° of physical activity score at 3 months after surgery, Y-axis; cytokine levels in synovial fluid at 3 to 4 days after surgery

To examine if elevated levels of cytokines in synovial fluid in the acute phase after ACL reconstruction affected recovery status at 3 months post-surgery, acute phase cytokine levels were compared to patients’ 5-grade of physical activity scores at 3 months (Table 2). As indicated in Fig. 5, levels of IL1 beta significantly increased in the delayed recovery group (score 1–3) when compared to the quick recovery group (score 4–5) based on the patients’ physical activity at 3 months (*p* = 0.03). In addition, levels of IL6, IL10 and IFN gamma were also increased in patients with lower physical activity at 3 months (*p* = 0.08, respectively).

## Discussion

The most important findings of this study was that the severity of joint inflammation in the acute phase after surgery was significantly higher if the timing of index ACL reconstruction surgery was delayed. The physical activity recovery was greatly affected by the severity of acute joint inflammation in the early phase after surgery.

Previous studies have revealed several factors that influence the prognosis of ACL reconstruction surgery (Andernord et al. 2013; Czuppon et al. 2014; Feller & Webster 2013; Petersen et al. 2014). Among these factors, timing of surgical treatment after ACL injury is still controversial (Krutsch et al. 2015; Kwok et al. 2013). In this study, we divided patients into two groups according to the preoperative periods of 60 days. Patients are assumed to be able to walk easily and navigate up/down stairs with or without difficulty by 60 days after injury on our rehabilitation protocol after ACL reconstruction. Therefore, 60 days may represent a reasonable time point to determine the indication of an acute ACL surgery after injury (Andernord et al. 2013). In this study, we focused on the short period physical recovery because the early recovery status significantly affects the recovery to return to sports in the long run. The results showed that the physical activity at 3 months after ACL reconstruction in Group II was significantly lower than that in Group I. A systematic review reported that among the studies searched (49 studies in total), 39 % of studies permitted running at 3 months (Harris et al. 2014). The results of the current study would provide a basic biologic background for the review. Andernord et al. (Andernord et al. 2013) showed patients that underwent reconstruction at a sub-acute stages (2–12 weeks) after ACL injury had higher activity levels after 2–2.5 years than those who underwent delayed surgery by 12–24 months. We need to follow up the patients of this study to find out the relationship of the cytokine levels in joint fluid 3, 4 days postoperatively and the long-term recovery after ACL reconstruction between the Group I and Group II.

TNF alpha was significantly increased in the patients of Group II (Fig. 3). Patients with a delayed recovery rate at 3 months after surgery had higher levels of the inflammatory cytokines IL1 beta, IL6, and IFN gamma at 3 to 4 days after surgery (Fig. 5) although it is unclear why cytokine levels were increased in patients with a longer preoperative period (Group II). In addition to the pro-inflammatory cytokines (TNF alpha, IL1 beta, IL6 and IFN gamma), levels of an anti-inflammatory cytokine, IL10, in the synovial fluid at 3 to 4 days after surgery were also significantly increased in the patients of Group II (Fig. 3) and in patients with delayed physical activity recovery at 3 months (Fig. 5) (Schulze-Tanzil et al. 2009). John et al. and other groups indicated an increase in not only the pro-inflammatory cytokines such as TNF alpha, IL1 beta, and IL6 but also the immuno-regulatory cytokine IL10 after tendon injury, although the role of IL10 on tenocytes remained unclear (John et al. 2010). The results of this study regarding IL-10 showed similar trend. IL10 is an immuno-regulatory cytokine that can downregulate active immune responses and acts mainly on T cells or macrophages to attenuate inflammatory cytokine production and antigen presentation. IL10 deficient mice develop spontaneous colitis, suggesting the important roles of IL10 in regulating overactive inflammatory responses (Gomes-Santos et al. 2012). Physiological functions of IL10 during the healing process after ACL reconstruction surgery has yet to be elucidated, however, our data suggest that the appropriate inflammatory response greatly affect the recovery process after surgery.

A limitation of the current study was that it was performed retrospectively without reasonable control. Also, it was not investigated whether the short-term outcome affects the longer-term clinical outcome of the patients of the study. Future studies will include the investigation of longer-term outcomes of the patients in the current study, the investigation of whether the elevated cytokines in the patients of Group II have an effect on the progress of osteoarthritic changes during longer- term follow-up, and the investigation of modifications in the early phase after surgery to improve the early phase outcome and the longer-term results after ACL reconstruction.

## Conclusion

Delayed anterior cruciate ligament reconstruction surgery increases inflammatory cytokine levels in synovial fluid after surgery and hinders short-period recovery of the patients.

## Abbreviations

ACL: Anterior cruciate ligament
IFN: Interferon
IL: Interleukin
TNF: Tumor necrotic factor.
